# Global health training in paediatric residency programs: the Italian experience

**DOI:** 10.1186/1824-7288-41-S2-A62

**Published:** 2015-09-30

**Authors:** Daniele Roncati, Salvatore Aversa, Andrea Bon, Alessandro Mazza, Davide Vecchio, Liviana Da Dalt

**Affiliations:** 1 Osservatorio Nazionale Specializzandi Pediatria (ONSP), Padua, Italy; 2 Department of Paediatrics, University of Padova, Padua, Italy

## 

Osservatorio Nazionale Specializzandi Pediatria (ONSP) is an Italian association of residents in paediatrics, and one of its interests is to support paediatric training in developing countries. In 2014, for this purpose, ONSP performed a survey with the aim of describing interest, participation, resources, and obstacles of residents who are involved in global health training within paediatric residency programs. Once the final data were known, an informative brochure was produced for publicizing the results of this survey and the projects that Italian Pediatric Schools have activated in developing countries.

35 of 38 paediatric residency schools (92%) participated in the survey. 67% of them offer an elective training program in global health and 42% have a formal program that is part of the curriculum of trainees. 47% of the paediatric residency schools have a collaborative program with developing country or non-governmental organization (NGO) and 17% had a program in the past years but not still ongoing.

In most cases, 3^rd^, 4^th^ and 5^th^ year paediatric trainees were involved in “global health” training. The duration of training was less than 1 month in 13% of cases, 1-3 months in 39%, 3-6 months in 43%, and more than six months in 4%.

Only 22% of residents who were involved in training in developing countries were evaluated before departure, and only 35% received training in global health before leaving; 39% participated in post elective debriefing meetings.

74% had the supervision of a local tutor (66% pediatricians, 34% other doctors) and 61% also had an Italian tutor for the whole project.

Almost all of the collaborative programs were in Central or Southern Africa; only two of them were in Central America (Nicaragua).

The training in global health provided child care (in 62% of cases), neonatal care (45%), malnutrition support (58%), HIV prevention and AIDS care (20%) and local staff training (42%).

All residents were satisfied after their training in developing countries and only a few of them suffered for some minor reasons during that period.

In conclusion, more than 50% of Italian paediatric residency programs actually offer an elective program of global health program and most of the trainees consider it a great opportunity for professional growth.

